# Reduced fear conditioning after viral vector mediated neuropeptide Y administration into the basolateral amygdala

**DOI:** 10.1186/1471-2210-11-S2-A3

**Published:** 2011-09-05

**Authors:** Dilip Verma, Ramon O Tasan, Mario Mietzsch, Stefan Weger, Regine Heilbronn, Herbert Herzog, Günther Sperk

**Affiliations:** 1 Institute of Pharmacology, Medical University Innsbruck, 6020 Innsbruck, Austria; 2 Institute of Virology, Charité, Campus Benjamin Franklin, Free University of Berlin, 12203 Berlin, Germany; 3 Neuroscience Research Program, Garvan Institute of Medical Research, Darlinghurst NSW 2010, Australia

## Background

Neuropeptide Y (NPY) is a 36-amino-acid peptide that is abundantly expressed in the central nervous system. It is involved in various physiological and pathophysiological processes, including energy homeostasis, pain and epilepsy, but also in anxiety and depression. Consistent findings have demonstrated an anxiolytic effect of NPY. The presence of different NPY receptors in the amygdala and the effects of NPY on anxiety raise the question, whether NPY and its receptors may influence acquisition and extinction of conditioned fear. Therefore, we investigated NPY and NPY receptor knockout mice in Pavlovian fear conditioning.

## Methods

Pavlovian fear conditioning is a simple form of associative learning. NPY knockout (NPY-KO) mice as well as Y receptor knockout mice (Y_1_, Y_2_, Y_4_ and Y_1_/Y_2_ double KO) were subjected to a discriminative delay fear-conditioning paradigm. Extinction learning was performed the following day by repetitive exposure to the tone in the absence of a foot shock.

## Results

In cued fear conditioning NPY-KO mice acquire higher freezing levels and show increased expression and impaired extinction of conditioned fear. Y_1_-KO mice show faster conditioning and delayed extinction, whereas Y_2_-KO mice are similar to wildtype mice. Compared to Y_1_-KO mice, however, Y_1_/Y_2_ double KO mice exhibited enhanced fear acquisition and impaired between session extinction, indicating an important role of Y_2_ receptors in these processes. Interestingly, Y_4_-KO mice show normal fear conditioning but impaired extinction. Adeno-associated viral (AAV) vector-mediated over-expression of NPY in the basolateral amygdala (BLA) of NPY-KO mice significantly reduced the increased fear acquisition of NPY-KO mice. In addition, extinction was significantly improved after AAV-induced over-expression of recombinant NPY (rNPY) in the BLA of NPY-KO mice. No change was observed, however, after over-expression of rNPY in the central amygdala.

## Conclusions

Our data indicate that NPY has an inhibitory role in the acquisition and facilitates extinction of conditioned fear. These effects seem to be mediated predominantly in the BLA. In particular, the Y_1_ receptor may modulate the acquisition of fear, whereas for extinction a concerted action of Y_1_ and Y_4_ receptors seems to be conceivable.

